# Contact with psychiatric care prior to suicide: are there differences between migrants and the majority population in Sweden? A cohort study of 12 474 persons who died by suicide between 2006 and 2016

**DOI:** 10.1017/S2045796022000397

**Published:** 2022-07-27

**Authors:** E. Jonsson, H. Sjöqvist, M. Sundvall, S. Bäärnhielm, C. Dalman, A.C. Hollander

**Affiliations:** 1 Department for Global Public Health, Karolinska Institutet (KI), Stockholm, Sweden; 2 Department of Learning, Informatics, Management and Ethics (LIME), KI, Stockholm, Sweden; 3 Centre for Psychiatry Research, Department of Clinical Neuroscience, KI, Stockholm, Sweden

**Keywords:** Epidemiology, minority issues and cross-cultural psychiatry, psychiatric services, suicide

## Abstract

**Aims:**

The aim of this study was to determine possible differences in psychiatric care contact and the type of contact in the year prior to suicide by migrant status and region of origin compared to Swedish persons.

**Methods:**

A population-based open cohort design, using linked national registers, to study all individuals aged 20–64 years who died by suicide between 1 January 2006 and 31 December 2016 in Sweden (*N* = 12 474). The primary exposure was migrant status compared to the Swedish majority population in the following categories: non-refugee migrants, refugee migrants and children of migrants. The secondary exposure was region of origin in seven regions: Sweden, other Nordic countries, Europe, Sub-Saharan Africa, the Middle East and North Africa, Asia, the Americas and Oceania. The four outcomes were psychiatric in- and outpatient care, prescribed and purchased psychotropic medication and a variable composing the other variables, all measured the year before death. Logistic regression models adjusted for age, sex, income and marital status estimated the likelihood of psychiatric care utilisation by type of care within the year prior to death by migrant status and region of origin (individually and combined).

**Results:**

Out of all who had died by suicide, 81% had had psychiatric care of any type in the year before death by suicide. Among refugees the prevalence of psychiatric care before death by suicide was 88%. Compared with the Swedish reference group, non-refugees and persons from Asia and Sub-Saharan Africa had a lower likelihood of utilising psychiatric care prior to suicide driven by a lower use of prescribed psychotropic medication. Persons from the Middle East and North Africa had a higher likelihood, driven by higher use of psychiatric outpatient care and prescribed psychotropic medication. Non-refugees' likelihood of utilising care before death by suicide was lower within the first 5 years of living in Sweden.

**Conclusion:**

A large share of those who die by suicide use psychiatric care the year before they die. Non-refugee migrants and persons from Asia and Sub-Saharan Africa have a lower likelihood of utilising psychiatric care prior to suicide compared to Swedish, whereas persons from the Middle East and North Africa have a higher likelihood. Health care and policy makers should consider both migrant status, region of origin and time in the new country for further suicide prevention efforts.

## Introduction

Internationally, suicide is the 15th most common cause of mortality and the second leading cause among 15–29-year-olds globally. There are a range of effective suicide prevention measures, and psychiatric care is one of them (Saxena *et al*., 2014; Schaffer *et al*., 2016). Migrants in high-income countries have patterns of psychiatric care use different from populations born in the country, but this varies by gender, region of origin and time in the new country (Cooper *et al*., 2013; Abebe *et al*., 2017; Forte *et al*., 2018; Barnett *et al*., 2019; Hollander *et al*., 2020). Common barriers among migrants seeking psychiatric care includes language and communication, lack of knowledge or a mistrust of the healthcare system, or different expectations between providers and patients (Lokdam *et al*., 2016).

In recent years, Sweden has been one of the most accepting countries in the European Union for migrants, thus changing its demographics and possible healthcare need considerably (Nordregio, 2017). As of 2018, 19% of Swedish citizens were born abroad (Statistics Sweden, 2021). Migrants may face stressors stemming from the reason of migration such as war trauma (Tinghög *et al*., 2017), but also from stressors of trying to adapt to a new setting (Malm *et al*., 2020). How stressful that adaptation is depends upon the migrant's background as the more similar two cultures are the easier the adaption is (Nekby, 2012). Migration and adaptation stressors coincide with known risk factors for suicide, such as social and geographic isolation, low access to support and clinical care, unemployment and poverty (Saxena *et al*., 2014; Castañeda *et al*., 2015; Forte *et al*., 2018; Baldessarini, 2020). Migrants to Sweden initially have a lower risk of suicide, but with time in Sweden the risk of suicide increases (Björkestam *et al*., 2020; Hollander *et al*., 2019).

Sweden has a publicly funded universal health care system, still some groups of migrants in Sweden, especially from non-OECD countries, reported refraining from seeking psychiatric healthcare more than the native population (Hollander *et al*., 2020). Migrants from Sub-Saharan Africa, Asia, Western and Southern Europe use less psychiatric care in Sweden in comparison to Swedish born while migrants from the Middle East and North Africa use more (Hollander *et al*., 2020). In light of this inequality of psychiatric care utilisation by migrant status, there is a need of in-depth knowledge of what these inequalities consist of, and if they were present among persons who died by suicide. To our knowledge, no study has investigated contact and type of psychiatric care prior to suicide among migrants, by migrant status and region of origin as compared with the those born in the country. The aim of this study is to determine if there were differences in psychiatric care contact and the type of contact in the year prior to suicide by migrant status and region of origin compared to Swedish persons.

## Materials and methods

### Study population

The study population was an open cohort of all individuals ages 20–64 years who died by suicide between 1 January 2006 and 31 December 2016 in Sweden. The observational period begins when eligible persons turned 20, immigrated into Sweden or the study start, 1 January 2006. The inclusion criteria of this study were persons who died by suicide using ICD-10 codes X60-84 (for suicide) and Y10-34 (for deaths by undetermined intent) from the Cause of Death Register, a common practice in suicide research (Björkenstam *et al*., 2020; Hollander *et al*., 2019). Those without an official residence permit in Sweden i.e. undocumented migrants or individuals awaiting an official asylum decision are not included in this register linkage and thus not a part of this study population. Upon further examination of date of death, those with the month and day of death imputed were removed from the cohort (*N* = 121).

### Data sources

Individual data in the registers were linked using unique personal identity numbers and anonymised by Statistics Sweden for research purposes. The registers from Statistics Sweden were longitudinal integration database for health insurance and labour market studies (LISA), longitudinal database for studies of immigrants' integration (STATIV). The registers from the Swedish National Board of Health were the National Patient Register, Swedish Prescribed Drug Register and Cause of Death Register.

### Exposures

The primary exposure variable in this study will be referred to as migrant status. Migrant status was categorised as the Swedish majority population, non-refugee migrants, refugee migrants and children of migrants. The Swedish-born majority population comprised of Swedish born with at least one parent born in Sweden (from now on *Swedish*). Migrants all had a residence permit to stay in Sweden and were not born in Sweden, however, divided into two groups. One subgroup of migrants was refugee migrants (from now on *refugees*) and this group was migrants who had been granted to stay in Sweden for refugee reasons according to the STATIV. The other migrant group was non-refugee migrants (from now on *non-refugees*), and this group included all other kinds of migrants (such as labour migrants, family of labour migrants and family of refugees, etc.). Children of migrants were individuals that were born in Sweden with two migrant parents.

The secondary exposure was region of origin. To prevent misuse of data, Statistics Sweden does not record ethnicity or religion, and aggregates country of origin of migrants to Sweden into non-specific regions. Although Statistics Sweden does record data on specific country of birth, information is released for research purposes according to 13 larger geographical regions to ensure confidentiality. From this variable, we derived a broader region of origin variable for analysis, which included seven regions: Sweden, other Nordic countries, Europe, Sub-Saharan Africa, the Middle East and North Africa, Asia, the Americas and Oceania. Children of migrants' origin were based upon their parent's region of origin (maternal origin if parental origins differed) to represent cultural influence. This information was extracted from the LISA and STATIV database.

### Outcomes

The outcome in this study consists of contact with psychiatric healthcare within the year prior to death. The last contact date for each type of care was used. Type of care included psychiatric inpatient care: use of hospital psychiatric services; psychiatric outpatient care: specialist care in community psychiatric services; prescribed and purchased psychotropic medication (from now on prescribed psychotropics in text and prescription in tables). Psychotropic medications included are antidepressants (Anatomic Therapeutic Chemical [ATC] code N06A), attention deficit hyperactivity disorder (ADHD) medications (ATC N06BA01 to NO6BA04, N06BA09), antipsychotics (ATC N05A), anxiolytics (ATC N05A) and sedatives/hypnotics (ATC N05C). We also included a variable combining all care types called any care.

### Covariates

This study's socio-demographic factors include sex, age, civil status (unmarried, married, divorced, widow) and family disposable income (standardised in quintiles) and are derived from the LISA database. Civil status and income are measured on 31 December of the year before the index suicide. This ensures that the data are not compromised by the death of the individual. Missing data for these two covariates were coded as missing in each respective variable.

### Statistical analysis

Basic descriptive characteristics were stratified by migrant status (reported using counts and percentages) to show the distribution of covariates. The period prevalence of psychiatric care contact during the study period was differentiated by type of care among the exposure groups, migrant status and region of origin. The *χ*^2^ tests were conducted separately to test the association between migrant status and region of origin with type of psychiatric care. Logistic regression was used to calculate the odds ratio (OR) of psychiatric care utilisation by type of care within the year prior to death among migrants compared to Swedish as well as the effect of years lived in Sweden on that OR. The analysis included both exposure groups, to differentiate between migrant status and region of origin. The analyses were run separately due to the exposures sharing the reference group (i.e. Swedish). Analyses had two models. Model 1 was adjusted for sex and age as they are known confounders of both healthcare use and suicide. Model 2 adjusted for civil status and family disposable income in addition to age and sex. A nested likelihood ratio test tested whether sex had an interaction with migrant status on the multiplicative scale. A sensitivity analysis tested whether there was a difference in OR estimates based upon if the suicide was a known suicide i.e. suicide with known intent (ICD X60-84) or if the intent was unknown intent (ICD Y10-34). To test this, the analyses were re-run including suicide with known intent (ICD X60-84) only and compared with when cases with deaths by unknown intent (ICD Y10-34) were included to see if there was any effect of outcome and by this, see if there was possible misclassification.

### Ethical considerations

The proposed study used data from Psychiatry Sweden, which links multiple registers via personal identification numbers and is anonymised by Statistics Sweden. Data use from ‘Mental health, psychiatric disorders: occurrence and etiology’ has gotten ethical approval by the Stockholm Regional Ethical Review Board (number 2010/1185-31/5).

## Results

The cohort consisted of 12 474 persons that died by suicide between 2006 and 2016 (for detailed characteristics of the cohort, see Table 1). The cohort comprised of 82% Swedish born, the non-refugees predominantly originated from the Nordic region (35%) or Europe (33%) and the refugees were primarily from the Middle East and North Africa (56%). The largest share of suicides pertained to the lowest income quintile and among those unmarried. In Table 1, *χ*^2^ test by migrant status/region of origin, *p*-value < 0.05, were all significant.

**Table 1.** Descriptive characteristics of the cohort of persons who died by suicide in the years 2006–2016, by migrant status

Demographic factor	Swedish	Children of migrants	Non-refugee	Refugee
*N*	10 231	549	1512	182
Sex				
Female	2961 (28.9%)	147 (26.8%)	540 (35.7%)	37 (20.3%)
Male	7270 (71.1%)	402 (73.2%)	972 (64.3%)	145 (79.7%)
Age group				
20–29	1983 (19.4%)	143 (26.0%)	208 (13.8%)	32 (17.6%)
30–39	1776 (17.4%)	174 (31.7%)	271 (17.9%)	33 (18.1%)
40–49	2458 (24.0%)	139 (25.3%)	429 (28.4%)	47 (25.8%)
50–59	2943 (28.8%)	77 (14.0%)	437 (28.9%)	58 (31.9%)
60–65	1071 (10.5%)	16 (2.9%)	167 (11.0%)	12 (6.6%)
Civil status				
Married	1980 (19.4%)	66 (12.0%)	431 (28.5%)	51 (28.0%)
Unmarried	6141 (60.0%)	388 (70.7%)	628 (41.5%)	72 (39.6%)
Divorced	1962 (19.2%)	87 (15.8%)	409 (27.1%)	55 (30.2%)
Widowed	123 (1.2%)	4 (0.7%)	22 (1.5%)	4 (2.2%)
Missing	25 (0.2%)	4 (0.7%)	22 (1.5%)	0 (0.0%)
Disposable income				
0–19	2639 (25.8%)	204 (37.2%)	566 (37.4%)	75 (41.2%)
20–39	2247 (22.0%)	132 (24.0%)	351 (23.2%)	37 (20.3%)
40–59	1869 (18.3%)	85 (15.5%)	223 (14.7%)	28 (15.4%)
60–79	1854 (18.1%)	70 (12.8%)	192 (12.7%)	26 (14.3%)
80–100	1597 (15.6%)	54 (9.8%)	158 (10.4%)	16 (8.8%)
Missing	25 (0.2%)	4 (0.7%)	22 (1.5%)	0 (0.0%)
Region of origin				
Sweden	10 231 (100.0%)	2 (0.4%)	0 (0.0%)	3 (1.6%)
Nordic countries	0 (0.0%)	317 (57.7%)	531 (35.1%)	0 (0.0%)
Europe	0 (0.0%)	152 (27.7%)	505 (33.4%)	35 (19.2%)
Middle East and North Africa	0 (0.0%)	37 (6.7%)	153 (10.1%)	102 (56.0%)
Sub-Saharan Africa	0 (0.0%)	11 (2.0%)	71 (4.7%)	18 (9.9%)
Asia	0 (0.0%)	12 (2.2%)	140 (9.3%)	15 (8.2%)
The Americas and Oceania	0 (0.0%)	18 (3.3%)	112 (7.4%)	9 (4.9%)
Time in Sweden				
Not a migrant	10 231 (100.0%)	549 (100.0%)	0 (0.0%)	0 (0.0%)
0–4	0 (0.0%)	0 (0.0%)	259 (17.1%)	28 (15.4%)
5–9	0 (0.0%)	0 (0.0%)	90 (6.0%)	10 (5.5%)
10–14	0 (0.0%)	0 (0.0%)	174 (11.5%)	34 (18.7%)
15–19	0 (0.0%)	0 (0.0%)	206 (13.6%)	91 (50.0%)
20+	0 (0.0%)	0 (0.0%)	783 (51.8%)	19 (10.4%)

When comparing the share of the population with who had had any contact, refugees were the group with the highest proportion of contact with psychiatric care (88%), while non-refugees had the least (79%) (see Table 2). The differences between non-refugees, children of migrants and Swedish were small when looking at any psychiatric care, but the differences widen when looking at different care types. Those from the Middle East and North Africa had the highest proportional share of psychiatric care contacts (87%) while those from Sub-Saharan Africa had the lowest (71%) (see Table 2). In Table 2, *χ*^2^ test between migrant status and type of care, *p*-value < 0.05, were all significant.

**Table 2.** Period prevalence of psychiatric care type among persons who died by suicide in the years 2006–2016 by migrant status and region of origin (95% confidence interval (CI))

	Any care		Inpatient		Outpatient		Prescription	
	*N*	% (95% CI)	*N*	% (95% CI)	*N*	% (95% CI)	*N*	% (95% CI)
Total	1056	81 (80–82)	6887	55 (54–56)	7416	59 (57–60)	8985	72 (71–73)
Migrant status								
Swedish	8231	81 (80–82)	5651	55 (54–56)	6083	59 (58–60)	7377	72 (71–73)
Children of migrants	467	85 (80–85)	334	61 (56–66)	360	66 (61–71)	419	76 (72–80)
Non-refugee	1198	79 (77–81)	795	53 (50–56)	849	56 (53–59)	1 048	69 (66–72)
Refugee	160	88 (83–93)	107	59 (50–68)	124	68 (60–76)	141	77 (70–84)
Region of origin								
Sweden	8233	80 (79–81)	5652	55 (54–56)	6085	59 (58–60)	7378	72 (69–75)
Nordic countries	698	82 (79–85)	499	59 (55–63)	495	59 (55–63)	608	72 (68–76)
Europe	559	87 (84–90)	349	50 (45–55)	401	58 (53–63)	498	72 (68–74)
Middle East and North Africa	255	87 (83–91)	179	61 (54–68)	204	70 (64–76)	232	80 (75–85)
Sub-Saharan Africa	71	71 (60–82)	52	52 (38–66)	55	55 (42–68)	63	64 (52–76)
Asia	126	75 (67–83)	87	52 (41–63)	95	57 (47–67)	103	62 (52–72)
The Americas and Oceania	114	82 (76–90)	69	50 (39–63)	81	58 (48–60)	103	74 (66–82)

Table 3 shows that migrant status influenced the likelihood of utilising psychiatric care the year before death by suicide. The likelihood was lower in the non-refugee group compared to the Swedish reference group for any psychiatric care, outpatient care and prescribed psychotropics in both model 1 and model 2 (OR for any psychiatric care for non-refugees OR 0.86, 95% CI 0.76–0.97). This lower likelihood was not seen in non-refugee migrants in terms of inpatient care where there were no differences in terms of migrant status between any of the groups in both models. Refugees had an increased likelihood of outpatient care with little difference between the models (model 2 OR 1.44, 95% CI 1.07–1.94). When comparing the likelihood of utilising psychiatric care the year before death by suicide depending on region of origin (see Table 3), migrants from Asia and Sub-Saharan Africa had a lower likelihood of any care and prescribed psychotropics in comparison to Swedish in model 2 whereas persons from the Middle East and North Africa had a higher likelihood of any care, outpatient care and prescribed psychotropics in comparison to Swedish in both models (model 2 any care OR 1.40, 95% CI 1.05–1.88). Migrants from Nordic countries had a lower likelihood of utilising outpatient care (OR 0.84, 95% CI 0.72–0.97). The likelihoods of having any psychiatric care utilised varied across covariates (model not shown) with increased use with age and income. Married persons had the lowest likelihood of utilising care across the different civil statuses. Males had decreased likelihood of utilising care compared to females. Interaction analysis between migrant status and sex showed no interaction and was kept out of the odds regression models.

**Table 3.** Odds ratio (OR) of any psychiatric care in total, psychiatric outpatient care, psychiatric inpatient care and prescribed psychotropic medication within one year before suicide, by migrant status and region of origin (95% confidence interval (CI))

Any care					
Migrant status	Model 1	Model 2	Region of origin model	Model 1	Model 2
Swedish	1	1	Sweden	1	1
Children of migrants	1.19 (0.98–1.45)	1.14 (0.93–1.39)	Nordic countries	0.97 (0.82–1.13)	0.92 (0.78–1.08)
Non-refugee	0.88 (0.78–0.99)	0.86 (0.76–0.97)	Europe	0.97 (0.82–1.16)	0.97 (0.81–1.16)
Refugee	1.36 (0.96–1.91)	1.30 (0.92–1.84)	Middle East and North Africa	1.49 (1.13–1.96)	1.40 (1.05–1.85)
			Sub-Saharan Africa	0.63 (0.42–0.95)	0.62 (0.41–0.94)
			Asia	0.72 (0.51–1.01)	0.71 (0.50–0.99)
			The Americas and Oceania	1.03 (0.71–1.49)	1.02 (0.70–1.48)
*Outpatient*					
Swedish	1	1	Sweden	1	1
Children of migrants	1.11 (0.93–1.33)	1.06 (0.89–1.26)	Nordic countries	0.88 (0.76–1.02)	0.84 (0.72–0.97)
Non-refugee	0.90 (0.80–1.00)	0.88 (0.79–0.99)	Europe	1.04 (0.89–1.22)	1.05 (0.89–1.23)
Refugee	1.48 (1.10–1.99)	1.44 (1.07–1.94)	Middle East and North Africa	1.52 (1.20–1.93)	1.44 (1.14–1.86)
			Sub-Saharan Africa	0.83 (0.55–1.24)	0.80 (0.53–1.21)
			Asia	0.74 (0.54–1.01)	0.74 (0.54–1.02)
			The Americas and Oceania	0.98 (0.69–1.38)	0.97 (0.69–1.37)
*Inpatient*					
Swedish	1	1	Sweden	1	1
Children of migrants	1.11 (0.93–1.33)	1.05 (0.87–1.25)	Nordic countries	0.98 (0.84–1.14)	0.92 (0.79–1.07)
Non-refugee	0.92 (0.83–1.04)	0.90 (0.80–1.01)	Europe	0.89 (0.75–1.04)	0.88 (0.74–1.04)
Refugee	1.13 (0.83–1.54)	1.08 (0.79–1.47)	Middle East and North Africa	1.35 (1.07–1.72)	1.26 (0.99–1.60)
			Sub-Saharan Africa	0.99 (0.73–1.38)	0.96 (0.63–1.46)
			Asia	1.00 (0.73–1.38)	0.99 (0.72–1.37)
			The Americas and Oceania	0.87 (0.60–1.25)	0.85 (0.59–1.23)
*Prescription*					
Swedish	1	1	Sweden	1	1
Children of migrants	1.07 (0.90–1.29)	1.05 (0.87–1.26)	Nordic countries	0.89 (0.77–1.03)	0.87 (0.75–1.01)
Non-refugee	0.82 (0.73–0.91)	0.82 (0.73–0.92)	Europe	0.93 (0.79–1.10)	0.95 (0.80–1.12)
Refugee	1.15 (0.84–1.57)	1.15 (0.84–1.58)	Middle East and North Africa	1.33 (1.03–1.71)	1.30 (1.01–1.68)
			Sub Saharan Africa	0.60 (0.39–0.89)	0.61 (0.43–0.82)
			Asia	0.59 (0.43–0.81)	0.60 (0.43–0.82)
			The Americas and Oceania	0.87 (0.62–1.24)	0.89 (0.63–1.26)

Model 1 adjusted for age and sex. Model 2 adjusted for age, sex, civil status, family disposable income.

In Table 5 we evaluated the effect of years lived in Sweden on migrants' likelihood of using psychiatric care in the year before suicide. Non-refugee migrants that had been in Sweden for less than 5 years were less likely to use all types of psychiatric care (model 2 any care OR 0.48, 95% CI 0.37–0.63). Non-refugees' likelihood of prescription care after 20 years in Sweden was lower than Swedish at 0.78 (95% CI 0.66–0.91).

### Sensitivity analysis

The sensitivity analysis tested if there was a difference in results depending upon clear intent of suicide (X60-84) *v.* undetermined intent (Y10-34). When only including clear intent suicides (*n* = 9532, 76%), the likelihood and relative risk estimates were similar to original estimates, although less significant (see Table 5 in Appendix).

## Discussion

The aim of this study was to determine if there were differences in psychiatric care contacts and the type of contact in the year prior to suicide by migrant status and region of origin compared to Swedish persons. Compared with Swedish, non-refugees and persons from Asia and Sub-Saharan Africa had a lower likelihood of utilising care prior to suicide. Non-refugees' likelihood of utilising care was lower within the first 5 years of living in Sweden. Migrants from the Middle East and North Africa had a higher likelihood.

### Strengths and limitations

There are some major strengths to this study. The study includes the entire population that died by suicide in Sweden during this study period. The use of register-based data allows for a comprehensive examination of psychiatric care use before death, through several points of entry (in and outpatient care and the use of psychotropic medication). These two factors make these findings generalisable to its population with strong internal validity. The use of an open cohort with a long follow-up time (2006–2016) lends to a representative sample of the dynamic flow of migration, relevant to real time influxes of migration. It is of note, however, that this open cohort method results in different groups of immigrants. There is therefore a variation and heterogeneity within each migrant group. The time in Sweden analysis (Table 4) separates and evaluates the heterogeneity of migrants based upon time in Sweden but may also distinguish varying circumstances of access to care. Non-refugees' lower likelihood in the first 5 years in Sweden may indicate a general barrier to care in early years or specifically of non-refugees who came to Sweden in the 2015 migration wave. Dividing the already small refugee group however showed no significant results.

**Table 4.** Odds ratio (OR) of psychiatric care type within one year before suicide, given migrant status and years in Sweden (95% confidence interval (CI))

		Type of psychiatric care			
Migrant status	Years in Sweden	Any care (OR)	Outpatient (OR)	Inpatient (OR)	Prescription (OR)
Non-refugee	0–4	0.47 (0.37–0.61)	0.57 (0.43–0.74)	0.64 (0.48–0.85)	0.56 (0.43–0.72)
	5–9	0.87 (0.55–1.38)	0.80 (0.52–1.23)	0.82 (0.53–1.29)	1.02 (0.65–1.60)
	10–14	1.10 (0.78–1.55)	0.93 (0.68–1.26)	0.80 (0.57–1.10)	1.12 (0.81–1.55)
	15–19	1.18 (0.86–1.62)	1.21 (0.92–1.60)	1.31 (0.99–1.74)	1.04 (0.76–1.40)
	20+	0.96 (0.81–1.13)	0.96 (0.83–1.11)	0.98 (0.84–1.14)	0.79 (0.68–0.92)
Refugee	0–4	1.03 (0.45–2.37)	1.37 (0.65–2.92)	1.18 (0.55–2.53)	0.68 (0.32–1.45)
	5–9	4.01 (0.50–32.07)	2.90 (0.73–11.47)	1.87 (0.54–6.50)	1.49 (0.38–5.92)
	10–14	1.08 (0.52–2.21)	1.47 (0.74–2.89)	1.30 (0.65–2.60)	1.19 (0.59–2.39)
	15–19	1.32 (0.81–2.15)	1.42 (0.93–2.16)	1.12 (0.73–1.72)	1.25 (0.80–1.97)
	20+	2.52 (0.73–8.70)	1.39 (0.56–3.44)	0.56 (0.19–1.70)	1.42 (90.54–3.78)
Non-refugee	0–4	0.48 (0.37–0.63)	0.57 (0.43–0.75)	0.63 (0.47–0.84)	0.60 (0.47–0.78)
	5–9	0.84 (0.53–1.33)	0.76 (0.49–1.17)	0.78 (0.49–1.22)	1.03 (0.66–1.61)
	10–14	1.05 (0.74–1.49)	0.91 (0.67–1.24)	0.77 (0.55–1.07)	1.12 (0.80–1.55)
	15–19	1.15 (0.83–1.59)	1.20 (0.91–1.60)	1.29 (0.97–1.71)	1.04 (0.77–1.40)
	20+	0.93 (0.78–1.09)	0.93 (0.80–1.08)	0.95 (0.81–1.11)	0.78 (0.66–0.91)
Refugee	0–4	1.01 (0.44–2.34)	1.32 (0.62–2.83)	1.13 (0.52–2.43)	0.71 (0.33–1.52)
	5–9	3.66 (0.45–29.32)	2.66 (0.67–10.57)	1.69 (0.48–5.92)	1.46 (0.37–5.78)
	10–14	1.07 (0.52–2.22)	1.47 (0.74–2.91)	1.28 (0.64–2.59)	1.22 (0.61–2.46)
	15–19	1.27 (0.77–2.07)	1.39 (0.91–2.11)	1.07 (0.69–1.66)	1.24 (0.79–1.94)
	20+	2.29 (0.66–7.95)	1.27 (0.51–3.15)	0.50 (0.16–1.51)	1.37 (0.52–3.66)

Model 1 adjusted for age, sex.

Model 2 adjusted for age, sex, civil status, family disposable income.

This study also had other limitations. Apart from the three types of care, we have not considered psychiatric diagnoses in primary care, if the care was compulsory care or not, number of hospital days or outpatient unit visits – data that might have given an indication of the different needs of the persons but were not available in this dataset. Further, there is no indicator of type of mental illness or its severity that could affect the use of care. Severe diagnoses could either encourage psychiatric contact or be a barrier to reaching care. It is possible that some migrants may seek care out of country as well which may affect our estimates differentially. This study also does not have data on visits in primary care, yet all psychotropic medication dispensed through primary care is included.

### Comparing to other studies

This study only includes those that have died by suicide, while most other studies related to suicide examine risk or likelihood among an entire population group. As such, this study entails a specific population of persons, not representative of their whole migrant group and this should be kept in mind when comparing to other studies' findings. Among those who die by suicide, our findings are in accordance with the literature of psychiatric care utilisation being common within the year before death (Walby *et al*., 2018). As compared with studies on psychiatric care utilisation in general, this study confirms that some sub-groups, such as non-refugee migrants and migrants from Asia, that are in need of psychiatric care (as defined by later dying by suicide) still have a lower likelihood of being in contact with psychiatric care (Hollander *et al*., 2020). Just like other studies, we found that migrants from the Middle East and North Africa have a higher utilisation of psychiatric care in general. In accordance to our own findings, a study by Hollander *et al*. (2020) found that migrants that moved to Sweden before 2005 use more psychiatric care.

In our study refugees had a higher likelihood of outpatient care the year before death by suicide as compared to all other groups. This finding contradicts a recent study showing that refugees had a lower likelihood of psychiatric care utilisation in comparison to Swedish except if they were diagnosed with schizophrenia and stress-related disorders (Björkenstam *et al*., 2020) including Post traumatic stress disorder (PTSD). The possible presence of PTSD could explain the difference between this study's finding of high utilisation among refugees that die by suicide compared to studies concerning psychiatric care among all refugees. A study by Axelsson *et al*. (2020) found that unaccompanied refugee minors used more psychiatric care than young refugees arriving with their families. The authors speculated that this increased use among the unaccompanied refugee minors could be due to a higher prevalence of poor mental health and closer contact with Swedish society. In our study, migrants with the closest ties to the Swedish society, i.e. children of migrants, had similar levels of care to those with (at least one) Swedish-born parent.

### Interpreting this study

Health behaviour and illness perception are formed by socioeconomic position, cultural values, social norms and the acculturation process, which lends to diversity within and among migrant groups. As this study adjusted for socioeconomic position and the differences in estimates between models were small, this study suggests that socioeconomic position is not a large determinant. Hence explanations below will focus on cultural factors and structural determinants. The higher utilisation of psychiatric care among certain groups can be due to a higher accessibility to and/or a greater need of psychiatric care. Refugees may have a lower barrier to care in comparison to other migrants as this group potentially has more contacts with Swedish agencies due to for instance the asylum-seeking process and the organised refugee reception the first 18 months that possibly increases health literacy. The need for psychiatric care among refugees may also be greater (Tinghög, *et al*., 2017; Satinsky *et al*., 2019) partly due to mental illness and postmigration stressors, that is both more common in refugees than among other migrants (Tinghög *et al*., 2017; Malm *et al*., 2020) and known to increase the risk of suicide (Ásgeirsdóttir *et al*., 2018; Fox *et al*., 2021). All other migrant groups were less likely to have psychiatric care contacts than Swedish, especially in non-refugees' first 5 years in Sweden, which may suggest structural barrier to healthcare in this group. That migrant groups' likelihood differs depending upon care type may suggest that the barrier for some sub-groups is both contacting psychiatric care and differences in availability of certain channels/types of care.

In our study, all persons died by suicide, but the type of care before death varied between groups. It is unknown if this was due to the severity of illness presented when in contact with care or how it was interpreted by practitioners. Prescribed psychotropics in primary care without contact in specialised psychiatric care may be considered as the patient could be helped using a prescribed psychotropics but that either the general practitioner (GP) or the patient thought further specialist care was not needed. Outpatient care could be an indicator of less severe problems compared to inpatient care. Inpatient care is an indicator of severe illness where the psychiatrist thought that this person would not be able to manage the problems by themselves or with their social network. It can also be a sign of late help seeking in a suicidal process. Several studies suggest the likelihood of suicide differs depending upon what care a patient have been receiving, with inpatient care having the highest suicide risk after discharge (Haglund *et al*., 2019; Roos af Hjelmsäter *et al*., 2019; Baldessarini, 2020; Holländare *et al*., 2020). A study examining the deficiencies in care delivered to patients who died by suicide in Sweden found the most common deficiency to be between staff and patient and due to inadequate care, such as inadequate prescribing and suicide assessment (Roos af Hjelmsäter *et al*., 2019). In all types of psychiatric care (prescribed psychotropics, out- and inpatient care) there are many patient–GP/psychiatrist interactions where there can be misunderstandings in terms of illness severity, need for treatment and judging the capacity of available social support, especially if there is a cultural or language difference. A Swedish study found that the failure to understand the cultural language to communicate about suicide was a problem in the relation between clinicians and refugee women that had already attempted suicide (Sundvall *et al*., 2018). This study's high level of psychiatric care contacts in refugees who nevertheless die by suicide might point to the same communication and assessment problems including identification of depression as standard instruments may not adequately reflect the experience of depression (Haroz *et al*., 2017) or other disorders across cultures. Thus, suggesting the quality of care, not just access, needs to be further evaluated.

### Implications

A lower use of psychiatric care prior to suicide in certain groups is an indication of missed opportunities for individuals and evidence-based suicide prevention. Finding ways for these subgroups to use psychiatric care when in need of it is an important challenge. Refugees’ higher use of outpatient care shows that it is not inevitable that migrants in need of psychiatric care underutilise it and it would be of use to study what mechanism make this group find the way in. The development of understanding among clinicians on how suicidal communication may differ among multi-ethnic groups may help prevent further suicide (Kirmayer *et al*., 2011). Suggestions for further studies to evaluate the relationship between migrants and psychiatric care would be to consider differentiating frequency of care, factor in mental illness, to investigate what barriers are experienced in accessing psychiatric care, how patient provider communication may differ depending upon culture and migrant background and to perform qualitative studies on suicide autopsy in migrant groups.

## Conclusion

This all-encompassing study of persons who died by suicide found that non-refugee migrants and persons from Asia have a lower likelihood of utilising psychiatric care compared to Swedish, whereas persons from the Middle East and North Africa have a higher likelihood of utilising care before death by suicide. Health care and policy makers should consider both migrant status and region of origin for further suicide prevention efforts as well as cultural adaptation of care and suicide assessment for health equity.

## Data Availability

Data cannot be shared publicly under the terms and conditions of ethical approval for Psychiatry Sweden.

## Supplementary material

For supplementary material accompanying this paper visit https://doi.org/10.1017/S2045796022000397. click here to view supplementary material
