# Supplementary material for: Contact with psychiatric care prior to suicide: are there differences between migrants and the majority population in Sweden? A cohort study of 12 474 persons who died by suicide between 2006 and 2016
Source: Epidemiol Psychiatr Sci. 2022 Jul 27;31:e56. doi: 10.1017/S2045796022000397 (PMC9354119; doi:10.1017/S2045796022000397)
Supplement: Supplementary file 1 [file S2045796022000397sup001.docx]

Appendix: Table 5. Odds ratio (OR) of psychiatric care type within one year before intended suicide, given migrant status and region of origin (95% confidence interval (CI))

| **Any care** | | | | | |
| --- | --- | --- | --- | --- | --- |
| **Migrant status** | **Model 1** | **Model 2** | **Region of origin model** | **Model 1** | **Model 2** |
| Swedish | 1 | 1 | Sweden | 1 | 1 |
| Children of migrants | 1.13 (0.91-1.42) | 1.07 (0.86-1.35) | Nordic Countries | 0.87 (0.73-1.05) | 0.84 (0.70-1.01) |
| Non-refugee | 0.89 (0.78-1.02) | 0.87 (0.75-0.99) | Europe | 1.02 (0.84-1.24) | 1.00 (0.82-1.22) |
| Refugee | 1.17 (0.80-1.71) | 1.12 (0.76-1.64) | Middle East & North Africa | 1.54 (1.12-2.13) | 1.44 (1.04-1.99) |
|  |  |  | Sub Saharan Africa | 0.60 (0.37-0.96) | 0.60 (0.37-0.97) |
|  |  |  | Asia | 0.73 (0.50-1.05) | 0.70 (0.49-1.02) |
|  |  |  | The Americas & Oceania | 1.08 (0.71-1.65) | 1.06 (0.70-1.62) |
| **Outpatient** | | | | | |
| Swedish | 1 | 1 | Sweden | 1 | 1 |
| Children of migrants | 1.12 (0.91-1.38) | 1.06 (0.86-1.32) | Nordic Countries | 0.89 (0,75-1.06) | 0.85 (0.72-1.02) |
| Non-refugee | 0.94 (0.82-1.07) | 0.91 (0.80-1.04) | Europe | 1.07 (0.90-1.29) | 1.06 (0.88-1.27) |
| Refugee | 1.48 (1.04-2.10) | 1.42 (1.00-2.01) | Middle East & North Africa | 1.59 (1.20-2.11) | 1.50 (1.13-1.98) |
|  |  |  | Sub Saharan Africa | 0.82 (0.51-1.32) | 0.80 (0.49-1.30) |
|  |  |  | Asia | 0.80 (0.57-1.13) | 0.79 (0.55-1.11) |
|  |  |  | The Americas & Oceania | 0.96 (0.65-1.43) | 0.95 (0.64-1.41) |
| **Inpatient** | | | | | |
| Swedish | 1 | 1 | Sweden | 1 | 1 |
| Children of migrants | 1.10 (0.89-1.36) | 1.03 (0.83-1.28) | Nordic Countries | 0.92 (0.77-1.11) | 0.88 (0.73-1.05) |
| Non-refugee | 0.92 (0.81-1.06) | 0.89 (0.77-1.02) | Europe | 0.90 (0.74-1.09) | 0.87 (0.72-1.06) |
| Refugee | 1.03 (0.71-1.49) | 0.97 (0.67-1.41) | Middle East & North Africa | 1.39 (1.04-1.84) | 1.29 (0.97-1.71) |
|  |  |  | Sub Saharan Africa | 0.99 (0.60-1.60) | 0.95 (0.58-1.56) |
|  |  |  | Asia | 0.91 (0.64-1.30) | 0.90 (0.63-1.28) |
|  |  |  | The Americas & Oceania | 0.96 (0.64-1.44) | 0.93 (0.62-1.41) |
| **Prescription** | | | | | |
| Swedish |  | 1 | Sweden | 1 | 1 |
| Children of migrants | 1.10 (0.88-1.36) | 1.07 (0.86-1.33) | Nordic Countries | 0.88 (0.74-1.05) | 0.87 (0.73-1.04) |
| Non-refugee | 0.88 (0.77-1.01) | 0.88 (0.77-1.01) | Europe | 1.07 (0.89-1.29) | 1.08 (0.89-1.30) |
| Refugee | 1.10 (0.76-1.58) | 1.09 (0.75-1.56) | Middle East & North Africa | 1.41 (1.05-1.89) | 1.36 (1.01-1.84) |
|  |  |  | Sub Saharan Africa | 0.55 (0.34-0.87) | 0.56 (0.35-0.90) |
|  |  |  | Asia | 0.55 (0.34-0.87) | 0.63 (0.45-0.90) |
|  |  |  | The Americas & Oceania | 0.93 (0.63-1.38) | 0.94 (0.63-1.40) |
